# On the New Chemical Nomenclature

**Published:** 1802-12-01

**Authors:** 


### *On the New Chemical Nomenclature.*

THE nomenclature of the metallic salts is very defective in not expressing any distinction between the salts formed by the different oxydes of any metal combined with the same acid. The sulphat of iron, for instance, signifies indifferently a compound of sulphuric acid, and either of the oxydes of iron. It has been proposed to distinguish them by terming that salt which contains the metal more highly oxydated, an oxysulphat. This mode is, however, very objectionable. By it we can only distinguish salts formed by two different oxydes, whereas several of the metals are capable of several degrees of oxydation. It is particularly objectionable with respect to metallic muriats, for an oxymuriat properly signifies a salt containing oxymuriatic acid. We are by no means authorized to suppose it an indifferent matter, whether the super dose of oxygen be combined with acid or with the metal.* I should propose to distinguish the salts of different oxydes of the same metal, in the manner

---

* In which state of combination it exists in any salt, may be known if the exact proportion of the ingredients be accurately ascertained. For the quantity of oxygen must be exactly sufficient either to form one of the oxydes of the metal and no more, or it is in that state of combination for which the quantity exactly corresponds with what would be required.

that that the oxydes are themſelves diſtinguiſhed, that is, generally by either their order, (as 1ſt. 2d. 3d. oxyde) or by their colour, (as black, red oxyde.) The word oxyde may, in all caſes, be omitted without ambiguity, by which means the expreſſion may be ſhortened; and, in order to avoid the error that may be eaſily occaſioned by the great ſimilarity of the words ſulphat and ſulphite, &c. I ſhould propoſe to render theſe terms into the adjectives ſulphated and ſulphitous; in the ſame manner, phoſphat and phoſphite will form phoſphated and phoſpitous, &c.

The ſulphats of iron then may be expreſſed thus:

Sulphated black iron, — or ſulphat of black iron,

Sulphated red iron, — or ſulphat of red iron,

Signifyng the ſulphats formed by the black and red oxydes of iron.

The ſulphites of iron will be

Sulphitous black iron, — or ſulphite of black iron,

Sulphitous red iron, — or ſulphite of red iron.

Super and ſub ſulphats, &c. evidently may follow the ſame rule.

Mercury forms three different oxydes, the black, grey, and red. Whether any one acid will combine with all theſe oxydes, is, perhaps, not yet aſcertained. A nomenclature, however, upon general principles, muſt provide names for every poſſible combination. Muriats of this metal are an important claſs of ſalts, and ſuppoſing they may be formed with all the different oxydes, we ſhall have muriated black mercury, muriated grey mercury, and muriated red mercury.

The oxymuriats would be oxymuriated black, grey, and red mercury.

Oxydes diſtinguiſhed otherwiſe than by their colour, may be diſtinguiſhed in the ſame manner in their ſalts. Thus the 6th oxyde of antimony is called the acidulous oxyde, to diſtinguiſh it from the 5th, both being white. In the ſame manner, we may diſtinguiſh a ſalt formed by that oxyde; as,

Phoſphate of lime and acidulous antimony (James's powders.)

There is another ſet of ſubſtances, to which the ſame rule may be advantageouſly applied as in the ſalts. The ſulphurets, phoſphorets, and carburets of metals contain the baſes either combined with oxygen in different quantities, or in their pure metallic ſtate. The nomenclature has provided no diſtinction for theſe different compounds. I ſhould propoſe to diſtinguiſh them thus:

Sulphurets of Iron.

Sulphuret of bright iron (iron in the metallic ſtate)

———— black iron } black and red oxydes of iron.

———— red iron }

N n 4 Sulphurets

Sulphurets of Mercury.

Sulphuret of bright mercury.
——— black mercury.
——— grey mercury.
——— red mercury.

Hydrosulphurets and hydrogenated sulphurets would follow the same law. This last term, hydrogenated, badly expresses a combination of sulphurated hydrogen. It would properly signify a combination of hydrogen only. Hydrosulphurated sulphuret would accurately express a combination of sulphurated hydrogen and a sulphuret.

